# Computational evaluation of the biomechanical effects of position changes in the femoral neck system on Pauwels type III femoral neck fractures: an in silico study

**DOI:** 10.3389/fbioe.2025.1493555

**Published:** 2025-02-24

**Authors:** Xiang Zhang, Shenghang Zhang, Zhou Zhong, Wen Zhang, Zhongwei Xiong

**Affiliations:** ^1^ Department of Orthopaedics, West China Hospital, Sichuan University, Chengdu, Sichuan, China; ^2^ Clinical Medical College, Southwest Medical University, Luzhou, Sichuan, China; ^3^ Spinal Surgery, Xiangtan Central Hospital Spinal Surgery, Xiangtan, Hunan, China; ^4^ Department of Orthopedics, Luzhou Longmatan District People’s Hospital, Luzhou, Sichuan, China

**Keywords:** femoral neck fractures, femoral neck system, implant positioning, biomechanics, finite element analysis

## Abstract

**Introduction:**

Despite the biomechanical advantages of the Femoral Neck System (FNS), improvements in postoperative complication rates have not been significant. This study evaluated the effects of different FNS positions on the biomechanical stability of Pauwels type III femoral neck fractures (FNFs) using finite element analysis (FEA).

**Methods:**

Pauwels type III FNF models fixed with different FNS positions were constructed using various bolt lengths, bolt positions, and axis–bolt angles. Biomechanical parameters, including stiffness, maximum implant von Mises stress (MIVS), maximum interfragmentary shear stress (MISS), and maximum interfragmentary gap (MIG), were analyzed by simulating early postoperative weight-bearing. Entropy scoring was used to rank the performance of different fixation positions to determine the optimal FNS implantation position.

**Results:**

Compared with that of the standard model, the biomechanical stability changed when FNS positioning was altered. Among all the evaluated parameters, MIG had the highest weight (60.04%). In the lateral view, fracture fixation was most stable when the bolt was rotated 5° anteriorly relative to the femoral neck axis (composite score = 0.87). However, stability was poorer when the bolt was rotated 9° inward relative to the femoral neck axis (composite score = 0.13).

**Discussion:**

The MIG is an important biomechanical parameter for assessing the stability of different FNS positions when treating FNFs. Shortening the distance between the bolt and the subchondral bone, upward movement, external rotation, and anterior rotation of the bolt can help improve the stability of the FNS in the treatment of Pauwels III FNFs.

## Introduction

Internal fixation is a significant therapeutic approach for preserving the integrity of the hip joint in young and nondisplaced elderly patients with femoral neck fractures (FNFs) (Rajfer et al., 2024). However, owing to the near-vertical angulation of Pauwels type III FNFs, their substantial shear stress and inversion instability result in a high incidence of postoperative complications, such as fixation failure, malunion, and femoral head necrosis (Liporace et al., 2008). In response, DePuy Synthes (Johnson & Johnson Medical Devices, New Brunswick, NJ, United States) developed a new implant known as the Femoral Neck System (FNS) to treat young adult FNFs. It combines the advantages of the angular stability of dynamic hip screw (DHS) with the minimally invasive character of cannulated compression screws (CCSs) and has demonstrated excellent results in both clinical practice and biomechanical testing (Stoffel et al., 2017; Zhou et al., 2021). Despite the biomechanical advantages of FNS over DHS and CCSs, the incidence of postoperative complications remains unimproved and is not superior in improving final hip function and reducing postoperative pain (Rajnish et al., 2022; Kale et al., 2024). Therefore, we hypothesize that the change in the position of the FNS may explain this result.

**FIGURE 1:**
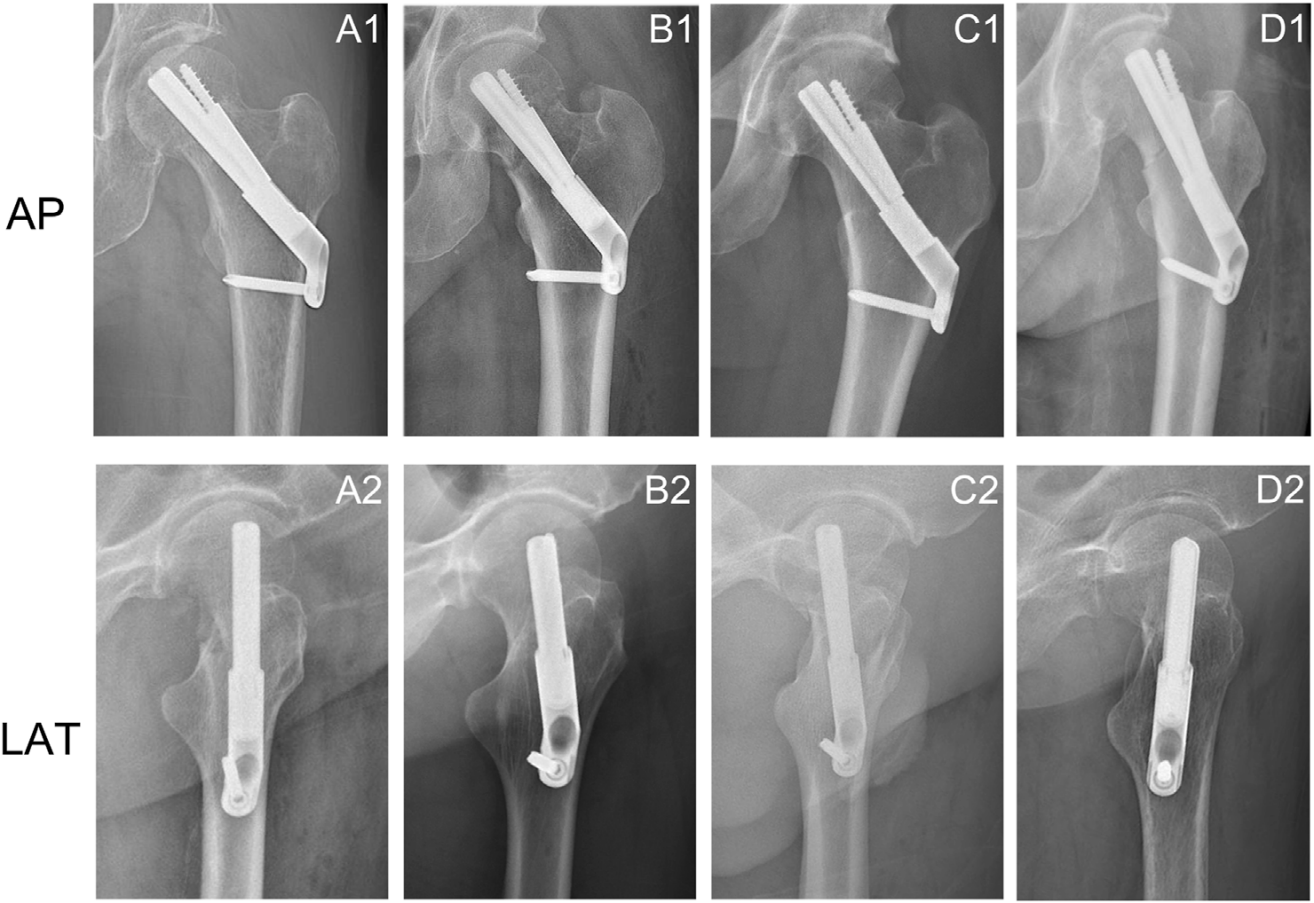
Anteroposterior and lateral X-rays of femoral neck fractures treated with FNS showed changes in the position of the FNS. **(A1, A2)** The bolt was implanted along the axis of the femoral neck in the anteroposterior and lateral views; **(A2, B2)** Change in bolt length; **(C1, C2)** Downward movement of the bolt in the anteroposterior view and posterior rotation of the bolt in the lateral view; **(D1, D2)** External rotation of the bolt in the anteroposterior views and anterior rotation of the bolt in the lateral view.

Researchers have identified bone mineral density, fracture type, quality of reduction, and implant position as risk factors for postoperative complications, such as fixation failure and malunion, in FNFs (Yang et al., 2013; Konstantinidis et al., 2016). During surgery, the position of the implant is one of the factors that the orthopedic surgeon can control. However, the optimal fixation position for FNS remains controversial. The manufacturers’ guidelines recommend inserting the FNS bolt along the femoral neck axis, but some scholars suggest placing the bolt below the femoral neck axis (Kuang et al., 2023). Different orthopedic surgeons rely mostly on their own clinical experience in determining the FNS position (Figure 1). Some biomechanical studies have also explored the effects of different FNS positions on the biomechanical characteristics of fixation stability (Jung et al., 2022; Nan et al., 2022). However, these studies have focused mainly on the bolt length and position in the femoral neck, with limited sample sizes, and studies that comprehensively assess the optimal position for FNS are lacking.

Finite element analysis (FEA) is an engineering mechanics method widely used in mechanical studies related to orthopedic surgery (Lewis et al., 2021). It possesses unique capabilities that are not available in other computational and experimental methods for calculating the complex mechanical behavior of the primary stability of fracture fixation structures (Schileo and Taddei, 2021). This study further refines the previous studies related to FNS positions and introduces a new measurement method, i.e., measuring the axis‒bolt angle (ABA) at the anterior-posterior (AP) and lateral (LAT) views as a complement to simulate the position of FNS in clinical practice. The effects of different FNS positions on the biomechanical stability of the fixed structure of Pauwels type III FNFs were also evaluated using FEA to explore the biomechanical mechanisms involved.

## Materials and methods

The mechanical experiments involved only image data from inpatients. The study protocol was reviewed and approved by the Biomedical Research Ethics Committee of our hospital (IRB #2021-1115).

### Finite element modeling

Computed tomography (CT) images of the left femur of a 26-year-old male weighing 70 kg were acquired using a CT scanner (SOMATOM Definition AS, SIMENS, Germany) with a layer thickness of 0.6 mm. The patient had no history of hip or systemic disease. The CT images were stored in digital imaging and medical communication (DICOM) formats and exported to Mimics 21.0 (Materialise Group, Leuven, Belgium) for 3D modeling. The 3D model of the left femur in STL format was imported into Geomagic Wrap 2021 (Geomagic, United States) for further surface smoothing, noise reduction, surface construction, and surface fitting and then output in STP. Finally, a model of the Pauwels type III FNF was generated in SolidWorks 2021 (DS Solidworks Corp., Waltham, MA, United States) using the “Cut” tool (a horizontal plane through the center of the femoral head was created, and then a straight line was drawn near the femoral neck at an angle of 70° to the horizontal line to cut the femoral neck).

**FIGURE 2:**
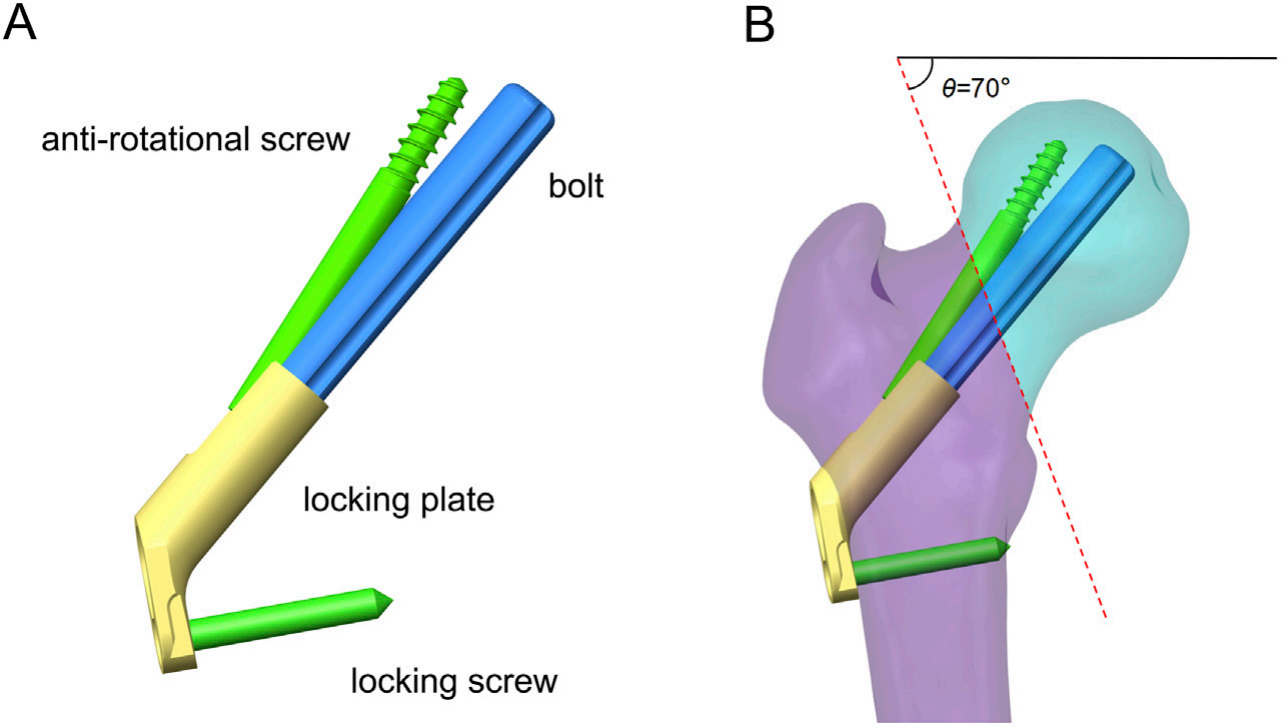
Schematic of FNS and fracture model. **(A)** Schematic diagram of FNS composition; **(B)** Standard Femoral Neck System model with 10 mm presliding for fixation of Pauwels type III femoral neck fracture.

On the basis of our previous study (Zhong et al., 2023), the 3D model of FNS was constructed in SolidWorks 2021 according to the dimensional information provided by the manufacturer. The single-hole FNS model consists of a single-hole locking plate, a locking screw, a bolt that slides freely on the locking plate, and an antirotation screw (Figure 2A). In addition, a standard FNS model with a 10 mm presliding bolt was constructed based on the presliding method described by Cha et al. (2023) (Figure 2B).

### FNS positioning

**FIGURE 3:**
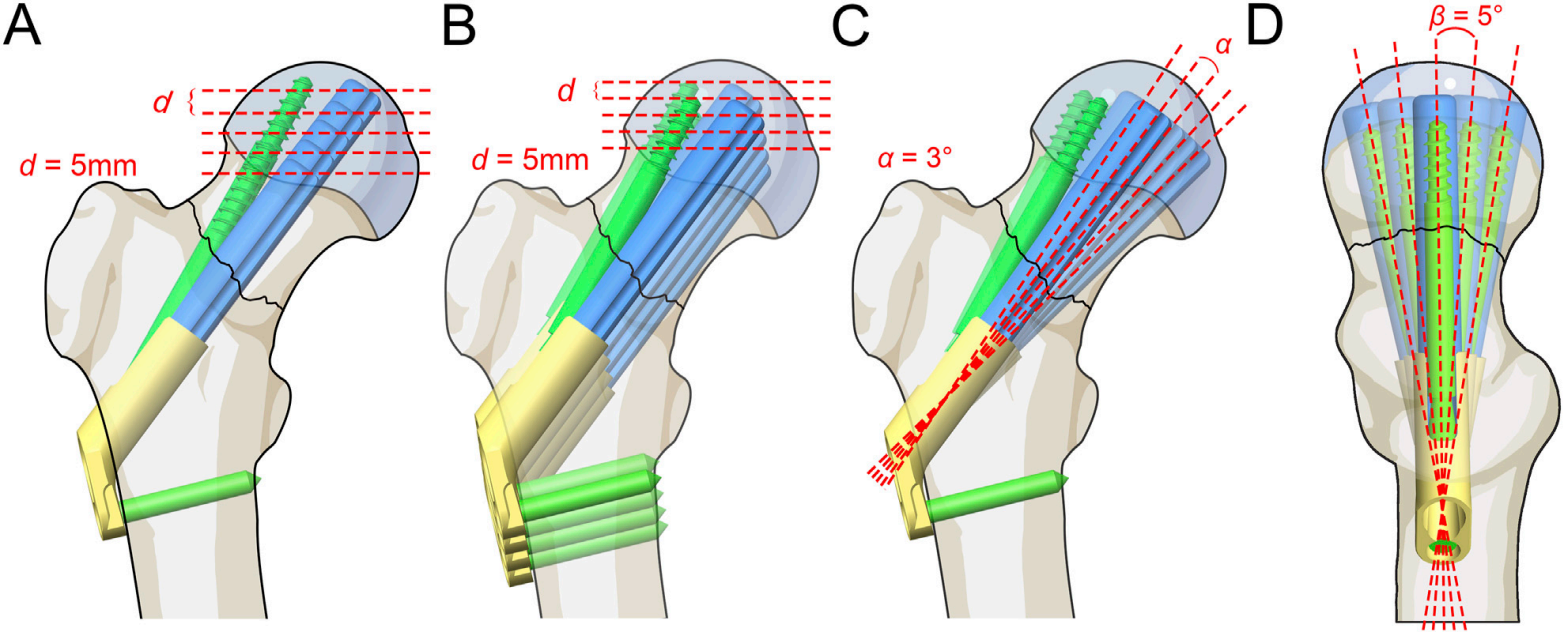
Schematic of different FNS positioning. **(A)** Bolt length variation; **(B)** Bolt position variation; **(C)** Bolt turning outward and inward; **(D)** Bolt rotates forward and backward.

**FIGURE 4:**
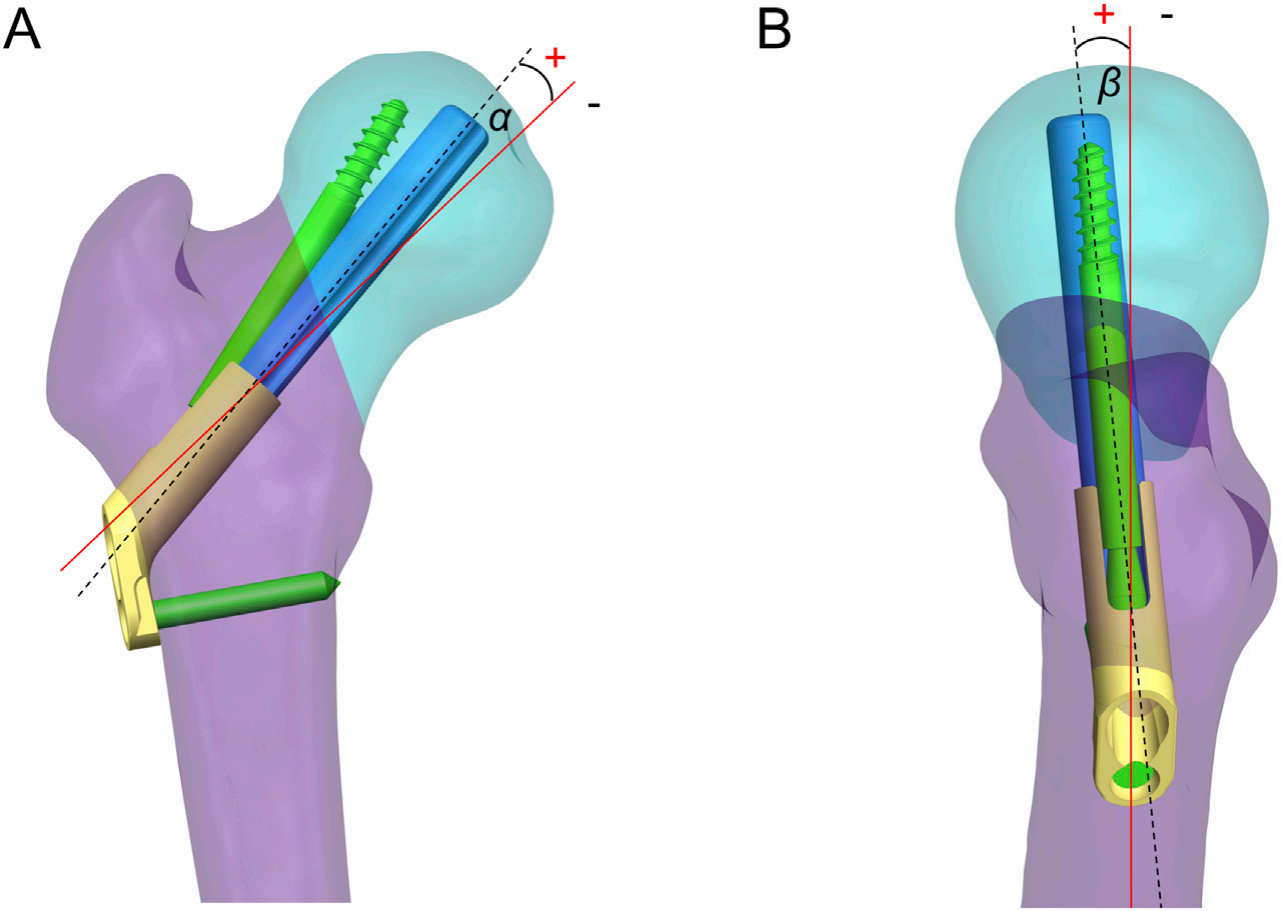
Axis-bolt angle (ABA) measurements. The angle between the femoral neck axis and the bolt was defined as the ABA, which is defined as α **(A)** and β **(B)** in the AP and LAT views.

The FNS model was virtually implanted into the FNF model using SolidWorks software. Seventeen Pauwels type III FNF models with different FNS positions were constructed. We subsequently determined the position of the femoral neck shaft using the Murphy method (Supplementary Figure 1). In the AP and LAT views, the bolt of the FNS was inserted along the femoral neck axis according to the manufacturer’s guidelines. Additional models were constructed on the basis of FNS positioning in clinical practice: 1) Bolt length variation (Figure 3A): the length of the bolt (75–95 mm) was changed with a variation of 5 mm based on the standard model; 2) Bolt position variation (Figure 3B): the bolt position was adjusted in 5-mm increments in the direction of the femoral shaft axis according to the standard model (positive for upward movement, negative for downward movement 5 mm to −15 mm); 3) Bolt turning outward and inward (Figure 3C): the angle between the femoral neck axis and the bolt was defined as the axis‒bolt angle (ABA), which is defined as α and β in the AP and LAT views, respectively (Figure 4). On the basis of the standard model, the bolt is turned in 3° increments to the femoral neck axis (positive for outward, negative for inward, α = 3° to −9°); and 4) Bolt rotates forward and backward (Figure 3D): on the basis of the standard model, the bolts are rotated anteriorly and posteriorly in 5° increments (positive for anterior rotation and negative for posterior rotation, β = 10° to −10°). Boolean operations were used to simulate bone loss during drilling and placement of the FNS in the surgical procedure (Jung et al., 2022).

### FE parameter setting

The established fixation models were imported into ANSYS workbench 2020R2 (Ansys, Canonsburg, PA) for further FEA. All the fracture models and FNS models were presumed to be composed of homogeneous and isotropic linearly elastic materials. The apparent femoral density (ρ), Young’s modulus (E), and Poisson’s ratio (v) of cortical and cancellous bone were calculated from the Hounsfield values in the CT images according to the following equations:

$$\rho \left(g/{\text{cm}}^{3}\right)=0.000968*\text{HU}+0.5$$

If ρ < 1.2 g/cm^3^, E = 2014 ρ ^2.5^ (MPa), ν =0.2.

If ρ > 1.2 g/cm^3^, E = 1763 ρ ^3.2^ (MPa), ν = 0.32.

The FNS model was defined as titanium alloy (Ti-6AL-7Nb) with Young’s modulus of 105 GPa, Poisson’s ratio of 0.34, and Yielding strength of 800 MPa (Jung et al., 2023).

Meshing was performed using a tetrahedral ten-node cell (C3D10) with a size of 1.5 mm on the basis of the results of mesh convergence experiments. The number of mesh elements (584615 - 641419) and nodes (749912 - 862640) varied with different solid models. The standard model was meshed at 1.0, 1.5, 2.0, and 2.5 mm in the mesh convergence analysis. The results showed that a mesh size of 1.5 mm produced a mesh-independent solution on the basis of the convergence of the single-leg standing load [changing the mesh size, the von Mises stress of the implant changed within 5% (Jyoti and Ghosh, 2022)]. Friction contact was defined as friction between the fracture ends and the bolt‒bone interface, with friction coefficients of 0.46 and 0.3, respectively. In addition, according to the design principle of FNS, the frictional coefficient factor for the interface between the bolt plate and antirotation screw plate was set to 0.2. Bonded contact was set up at the screw‒bone interface (Zhong et al., 2023).

**FIGURE 5:**
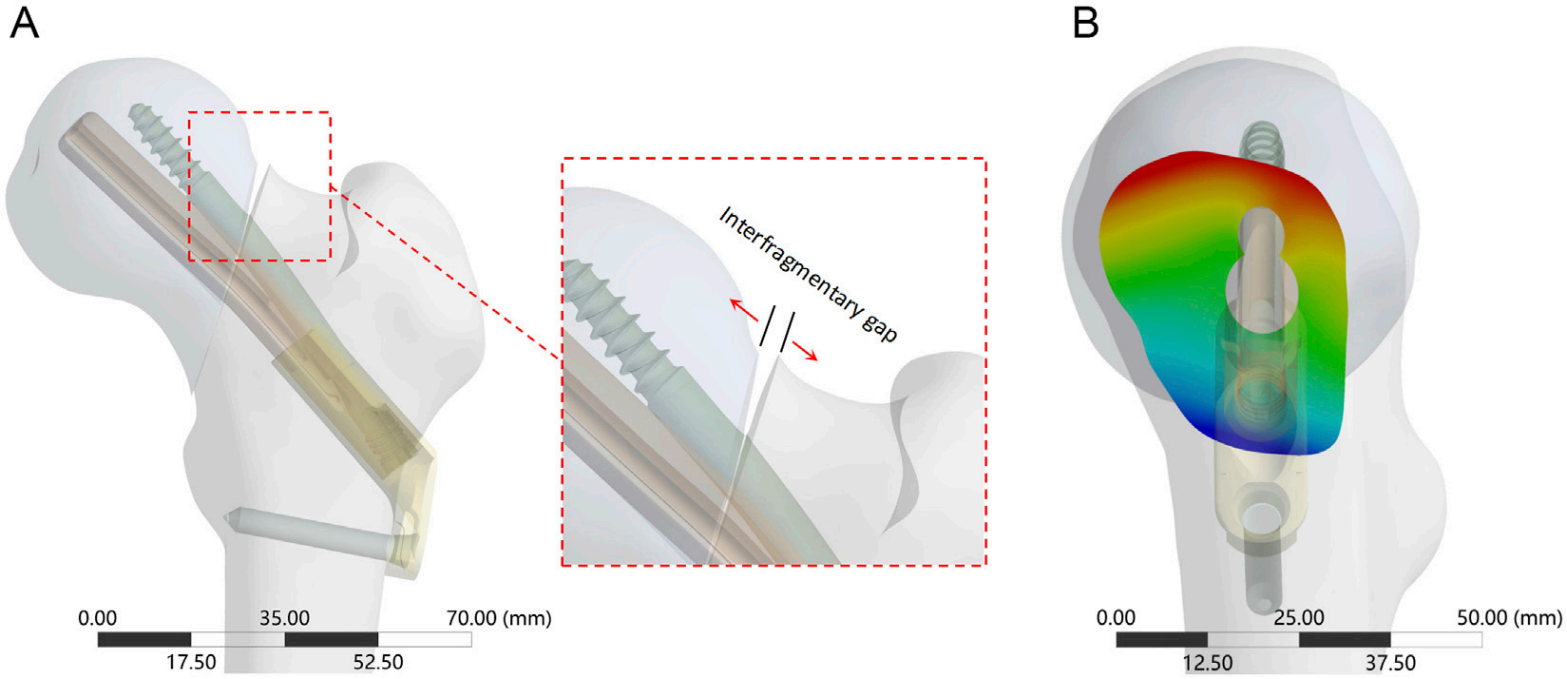
The interfragmentary gap between femoral head and femoral shaft fragments. **(A)** The enlarged view shows the direction of the detached displacement; **(B)** Cloud diagram of the fracture gap on the side view, with red deepening representing increased gap and blue deepening representing decreased gap.

Immediate postoperative fracture stability determines long-term stability (Bojan et al., 2018). This study simulated the loads on the femoral head when the patient stood on one leg after surgery. In accordance with Goffin et al. (2013), a new coordinate axis was established at the center of the femoral head to define the direction of the load (the force vector pointed posteriorly at an angle of 8° to the shaft in the sagittal plane and laterally at an angle of 13° to the axis of the femoral shaft in the coronal plane). The freedom of the distal femur was limited to 0. A force of 2100 N, equivalent to three times the body weight of a 70-kg patient (Huang et al., 2023a), was applied to the femoral head alongside the Z-axis of the new coordinate system. An extra 224 N preload was applied to simulate the compression effect of the FNS antirotation screw (Supplementary Figure 2) (Xia et al., 2021). The stability of the implants and fracture end was assessed by measuring the maximum implant von Mises stress (MIVS), maximum interfragmentary shear stress (MISS), and maximum interfragmentary gap (MIG, Figure 5) (Jung et al., 2022). In addition, the displacement of the fracture model in the load direction was evaluated to calculate the structural stiffness (the load divided by the displacement, representing the overall stability). The composite scores of the different FNS positions were subsequently ranked using an entropy scoring method to determine the optimal position (Zhan et al., 2024).

## Results

### Bolt length variation

**FIGURE 6:**
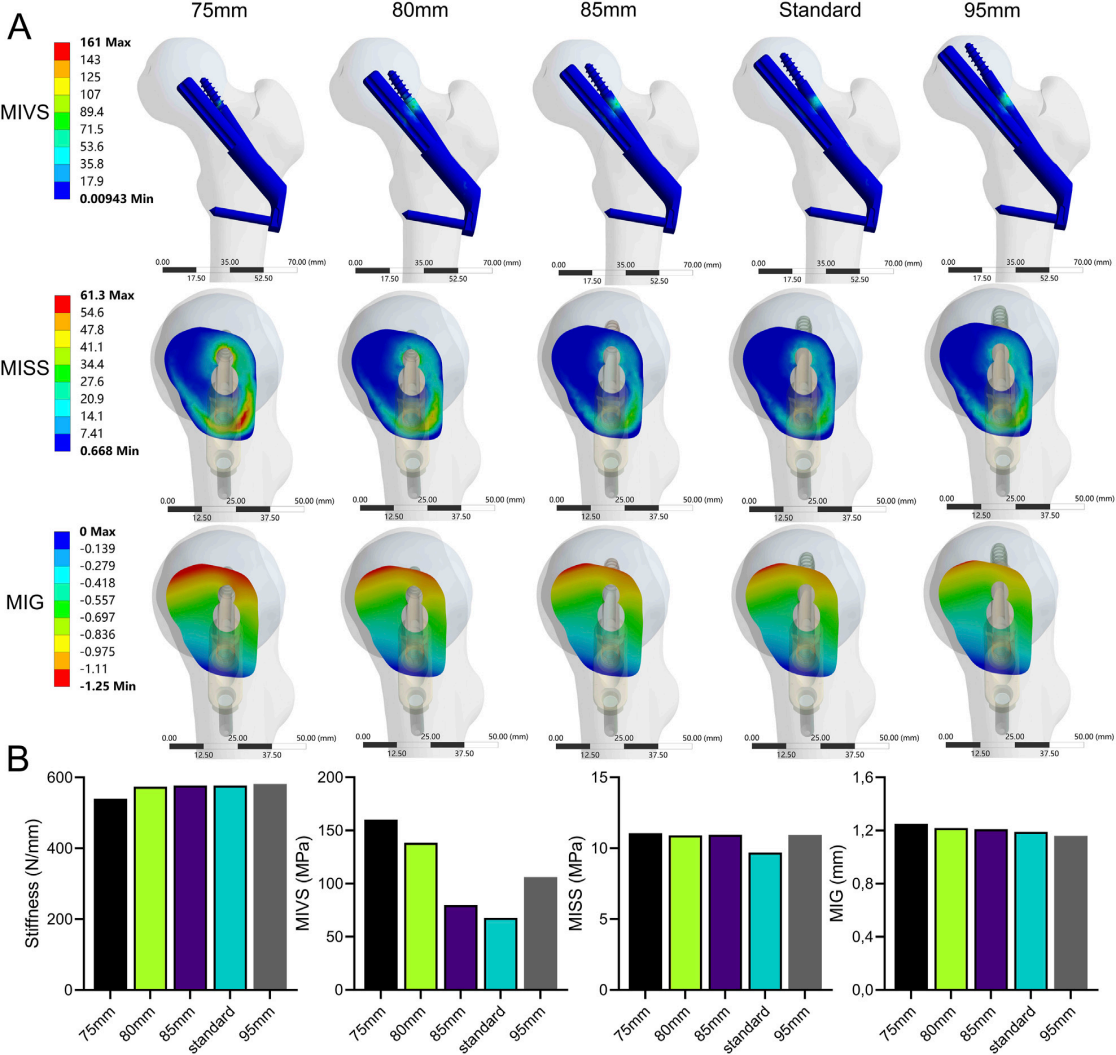
The cloud diagram **(A)** and histograms **(B)** of different FEA parameters obtained by varying the bolt length.

The stability of the fracture end decreased with decreasing bolt length (Figure 6; Supplementary Table 1). Compared with the standard model, the stiffness (539.85 N/mm) decreased by 6.5% when the bolt length was 75 mm. The MIVS (160.3 MPa) increased by 136.4% and was concentrated above the anti-rotation screw at the fracture line, and the MISS (11.07 MPa) increased by 14.1% and was concentrated at the anti-rotation screw‒bone contact surface and below the femoral neck. In addition, the MIG (1.25 mm) increased by 5% and was located above the femoral neck.

### Bolt position variation

**FIGURE 7:**
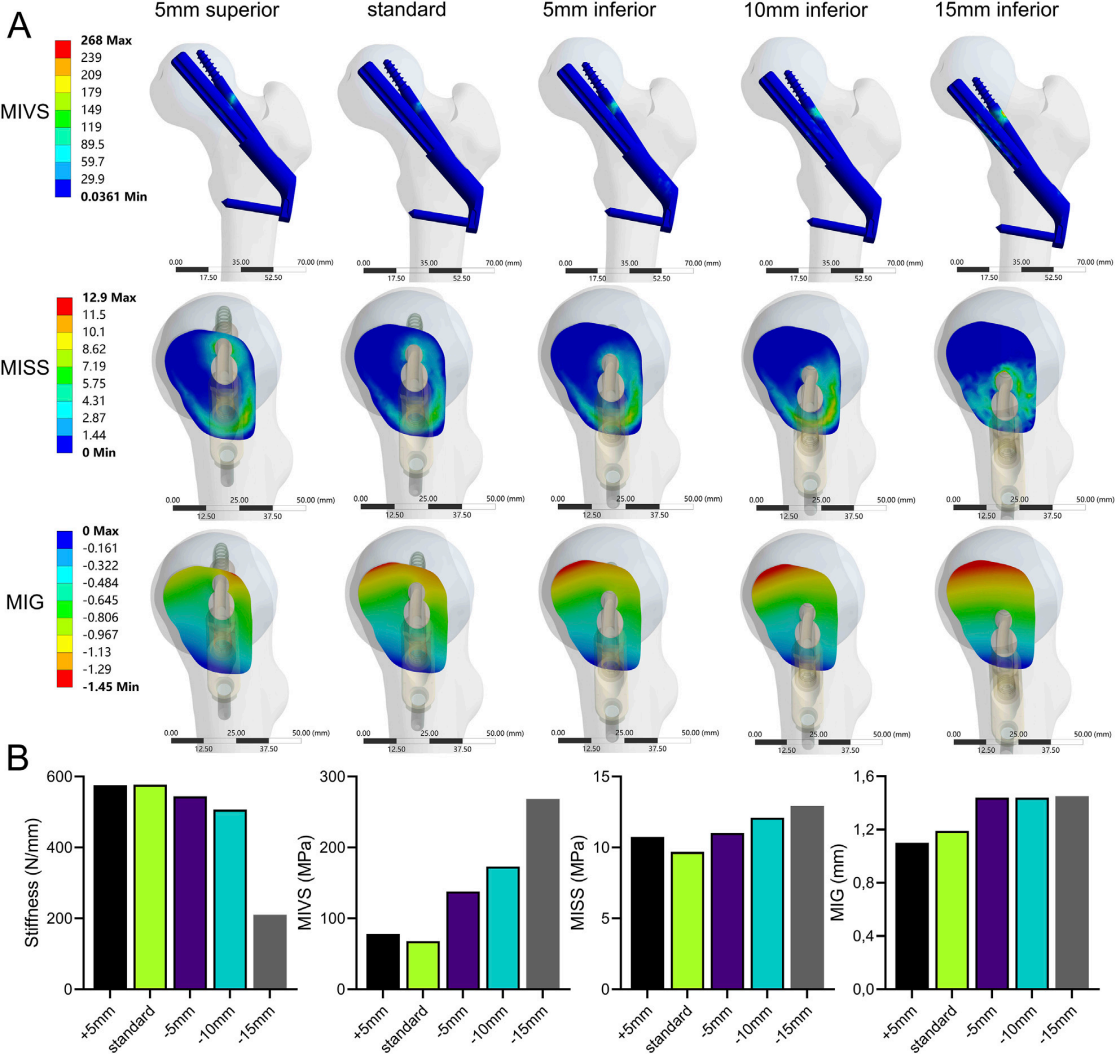
The cloud diagram **(A)** and histograms **(B)** of different FEA parameters obtained by varying the bolt length.

The stability of the fracture end decreased when the bolt was moved away from the femoral neck axis (Figure 7; Supplementary Table 1). Compared with the standard model, the strength was significantly lower when the bolt was moved 15 mm down the femoral shaft axis. This resulted in a 63.6% decrease in stiffness (210.2 N/mm) and a 295.9% increase in MIVS (268.4 MPa), concentrated above the anti-rotation screw at the fracture line. Additionally, MISS increased by 33.3% (12.93 MPa), with the stress concentration gradually shifting downward, and MIG (1.45 mm) increased by 21.8% and was located above the femoral neck.

### Bolts turned outward and inward

**FIGURE 8:**
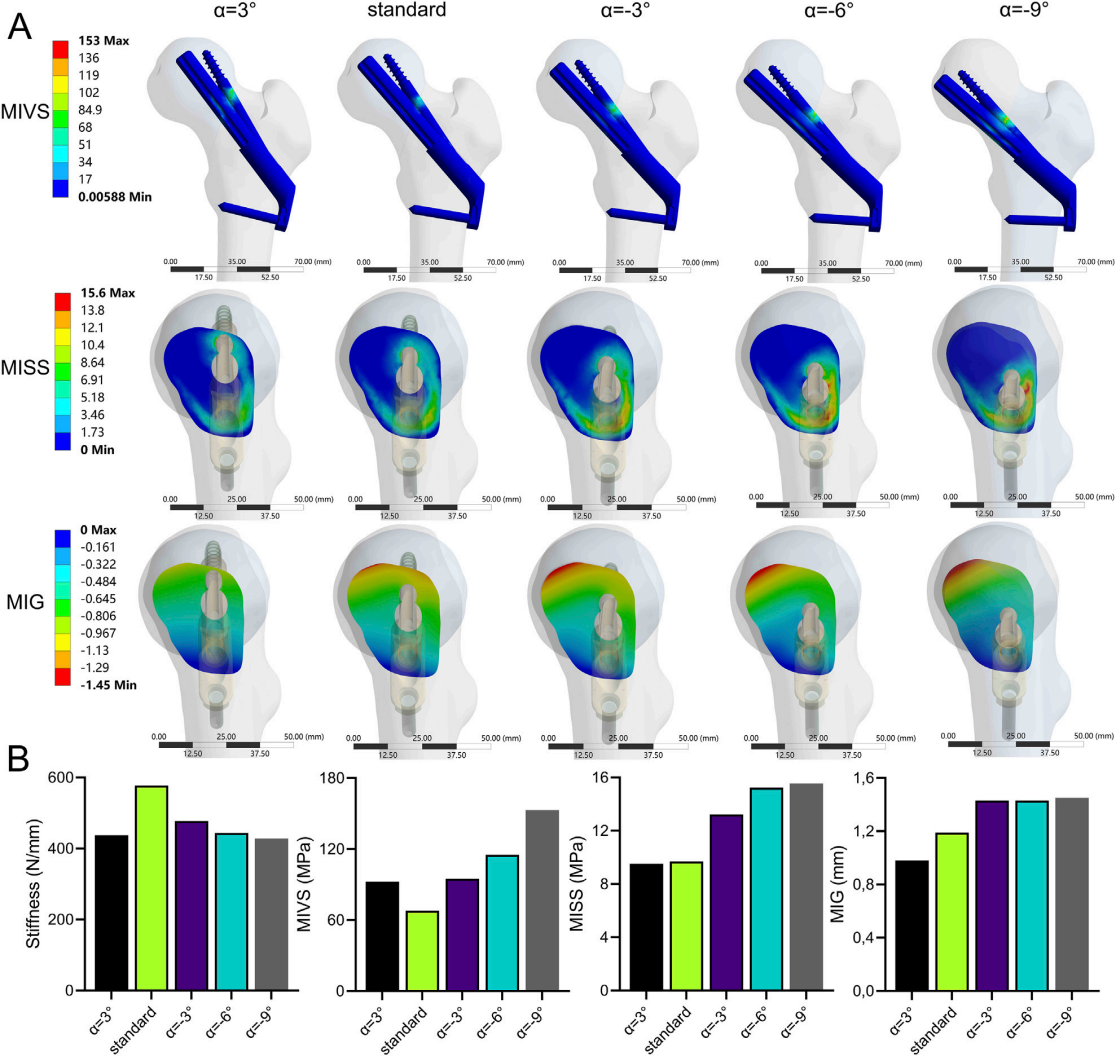
The cloud diagram **(A)** and histograms **(B)** of different FEA parameters obtained by varying the axis-bolt angle (α).

The stability of the fracture end decreased when the absolute value of ABA (α) increased (Figure 8; Supplementary Table 1). Compared with the standard model, the stiffness (427.93 N/mm) was significantly reduced by 25.9 at a 9° of inversion of the bolt (α = −9°). The MIVS (152.9 MPa) increased by 125.5% and was concentrated above the anti-rotation screw at the fracture line. The MISS (15.55 MPa) increased by 60.3% and was concentrated posteriorly below the femoral neck. The MIG (1.45 mm) increased by 21.8% and was located anteriorly above the femoral neck.

### Bolt rotation forward and backward

**FIGURE 9:**
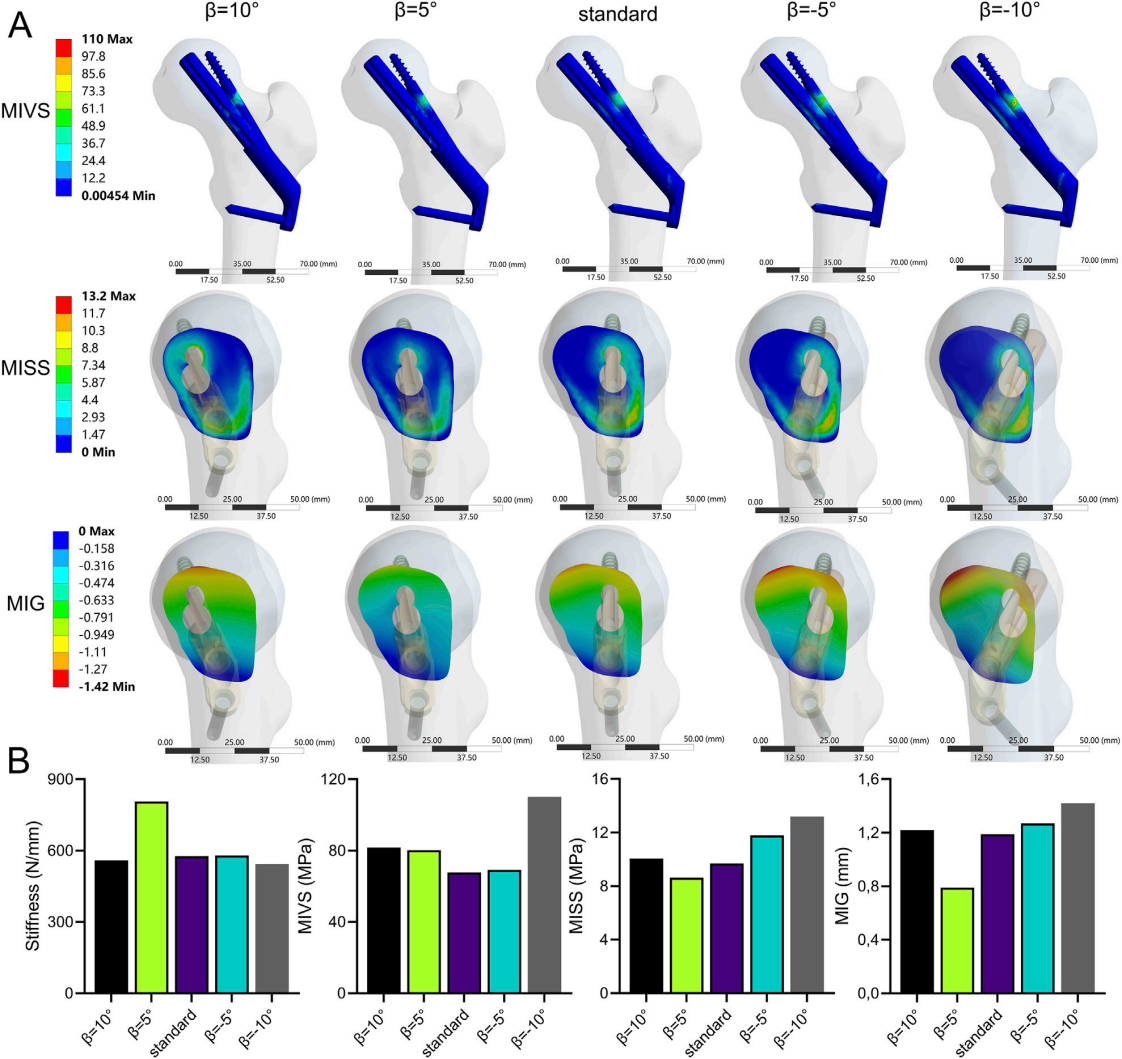
The cloud diagram **(A)** and histograms **(B)** of different FEA parameters obtained by varying the axis-bolt angle (β).

The stability of the fracture end increased and then decreased when the bolt was rotated anteriorly (β > 0°). In contrast, the stability of the fracture end decreased when the bolt was rotated posteriorly (β < 0°) (Figure 9; Supplementary Table 1). Compared with the standard model, the stiffness (805.7 N/mm) increased by 39.6%, the MISS (9.41 MPa) decreased by 3%, and the MIG (0.79 mm) decreased by 33.6% at β = 5°. However, when β = −10°, compared with the standard model, the stiffness (543.4 N/mm) decreased by 5.8%, and the MIVS (110 MPa) increased by 62.2%, which was concentrated above the anti-rotation screw at the fracture line. The MISS (13.2 MPa) increased by 36.1%, which was concentrated posteriorly below the femoral neck, and the MIG (1.42 mm) increased by 19.3%, which was located anteriorly above the femoral neck.

### Entropy score

The results of the entropy score method revealed that the information entropy values of stiffness, MIVS, and MISS were greater, the information utility values and the weighting coefficients (<20%) were lower, the information entropy value of MIG was lower, and the information utility value and weighting coefficient (>50%) were greater (Table 1). The weighting coefficients of the entropy values of each indicator were used to calculate the composite scores of the different FNS fixation positions. The results revealed that among the groups, the composite score of stability was greater when the screw was rotated forward by 5°, rotated outward by 3°, moved upward by 5 mm, and increased in length (Supplementary Table 1).

**TABLE 1:** Entropy value, utility value, and weight for fracture stability assessment parameters.

Parameters	Entropy value (e)	Utility value (d)	Weighting coefficient (%)
Stiffness	0.96	0.037	12.24
MIVS	0.97	0.029	9.78
MISS	0.95	0.054	17.94
MIG	0.82	0.18	60.04

MIVS, the maximum implant Von-Mises stress; MISS, the maximum interfragmentary shear stress; MIG, the maximum interfragmentary gap.

## Discussion

Owing to the high shear force caused by muscle traction, FNFs in young patients usually present as Pauwels type III fractures, with the fracture fragments displaced vertically (Sprague et al., 2015). Controversy remains regarding the biomechanical stability of different fixation methods for the treatment of FNFs. Several biomechanical studies have confirmed the mechanical superiority of FNS over CCSs and DHS in treating FNFs in young patients (Fan et al., 2021; Moon et al., 2022). However, the construction stability of FNS is weaker than that of CCS in the study by Xia et al. (2021). Additionally, the incidence of postoperative complications was not improved with the FNS compared with that with CCS fixation for the treatment of FNFs (Rajnish et al., 2022). During surgery, the FNS may be implanted in different positions. Therefore, we hypothesize that different FNS positions may change the biomechanical environment of the FNF, thereby increasing the incidence of postoperative complications such as fixation failure and nonunion. This study evaluated the biomechanical results of different FNS positions for the treatment of FNFs using FEA. Our primary results showed that among the biomechanical parameters, the MIG is an important indicator for assessing the initial stability of fracture fixation. In addition, the results of the composite score calculated using the entropy value score method for different FNS positions revealed that shortening the distance between the bolt and the subchondral bone, upward movement, external rotation, and anterior rotation of the bolt can help improve the stability of FNS for treating Pauwels III FNFs.

The FNS and Pauwels type III FNF models used in this study have been extensively studied using FEA (Jung et al., 2022; Ma et al., 2022). The stiffness of the FNS in this study (577.13 N/mm) was compared with the stiffness in the study by Huang et al. (2023b) (588.7 N/mm) and the finite element analysis performed by Xia et al. (2021) (593.22 N/mm). The results were consistent, verifying the effectiveness of the model construction method in this study and its suitability for further analysis. Changes in the biomechanical environment around the fracture end can affect healing. The structural stiffness, which reflects the overall stability of the fracture end, can be determined by calculating the displacement at the load application point (Samsami et al., 2015). The stress distribution map reflects the stress distribution in different parts of the implant under single-leg standing. The maximum implant stress is related to static yielding, a reliable predictor of fixation failure, and is widely used in biomechanical experiments (Cheng and Shoback, 2019; Lu et al., 2020). In addition, there is a variable gap at the fracture end, and the interfragmentary strain within the fracture gap is important in the fracture healing process (Elliott et al., 2016). Previous studies have shown that asymmetric lateral or axial movements hinder fracture healing (Elkins et al., 2016). Therefore, in this study, biomechanical indicators including stiffness, MIVS, MISS, and MIG were used to assess the stability of the fracture end.

When the bolt is inserted along the femoral neck axis, a short bolt results in reduced structural stability. The FNS is similar in shape to the DHS and has the characteristic of fixed-angle stability. Previous studies have shown that the tip‒apex distance determined by the DHS lag screw is a risk factor for fixation failure in treating intertrochanteric fractures (De Bruijn et al., 2012). The FNS also showed the same trend. Specifically, with increasing distance between the bolt and the subchondral bone, the composite score and fixation stiffness decreased, and the risk of fixation failure increased. However, further validation is needed to assess whether the same method can be used to evaluate whether the tip‒apex distance of the FNS is reasonable. Similar to the results of Jung et al. (2022), as the length of the bolt decreased, the MIVS, MISS, and MIG values increased, and the composite score of the model decreased. According to the lever-hinge theory (Budde et al., 2023), as the length of the bolt and the antirotation screw decreases, the distance between the reconstructed hinge of the FNS and the physiological hinge of the femoral neck increases, and the medial force arm increases, thereby increasing the risk of varus displacement. In addition, Cha et al. (2021) proposed a surgical technique to delicately control the FNS implant depth to achieve a smaller apex distance. Therefore, increasing the length of the bolt and shortening the distance between the bolt and the subchondral bone can effectively improve the stability of fracture fixation.

The distance between the bolt and the femoral neck axis is important for the stability of the fracture end. Jung et al. (2022) reported that inferior placement of the FNS is beneficial for fixation stability. However, this study revealed that when the bolt is aligned with the femoral neck axis, the MISS and MIG biomechanical indicators yield satisfactory results. When the bolt was placed above the femoral neck axis, the composite score was higher than that of the standard model, indicating increased fixation stability. In addition, when the bolt is placed below the femoral neck axis, the stiffness and the composite score of the model decreases with increasing distance between the bolt and the femoral neck axis, which is similar to the results of Kuang et al. (2023). This is because the vertical load of the hip joint is not transmitted through the femoral neck axis but rather through the inner edge of the femoral neck, which is subjected to greater tension (Bonneau et al., 2014). When the FNS bolts are placed inferior to the femoral neck axis, support above the femoral neck is lacking, leading to decreased fixation stability and a consequent increase in MIVS and MIG. Kuang et al. (2023) reported that when the FNS is placed below, a CCS can be used as a suitable revision option to increase fixation stability. Similarly, Siavashi et al. (2015) showed that adding a CCS above the DHS for the treatment of FNFs in the lower position can effectively improve fixation stability. Therefore, the FNS bolts should be inserted as close as possible to the femoral neck axis to ensure the stability of the fixation structure. In addition, when the FNS bolt is placed below the femoral neck axis during surgery, a CCS should be added above the FNS bolt as a remedial solution to increase the stability of fixation and reduce the degree of bone loss caused by repeated repositioning of the FNS bolt.

The ABA reveals the relationship between the bolt and the femoral neck axis in the AP and LAT views and is related to fixation stability. The results of this study showed that the stability of the bolt with outward implantation (α > 0) was significantly greater than that of the bolt with inward implantation. In particular, the combined fracture fixation score was significantly lower when α = −9°. With an increase in the MISS, the risk of cancellous bone fracture increases. Irreversible deformation of the cancellous bone occurs, resulting in fracture yielding (Panyasantisuk et al., 2016). However, when ABA (β) > 0°, fracture stability tended to increase and then decrease. The stiffness increases at 5° of anterior rotation of the bolt, which may be explained by the hip load transfer mechanism. Forces from the pelvis point posteriorly in the sagittal plane at an angle of 8° to the shaft axis (Bonneau et al., 2014), and the angular difference between the direction of the load and the bolt axis decreases with moderate anterior rotation, thereby increasing fixation stability. Similarly, Jung et al. (2023) reported that fracture stability was greater when the bolt was subjected to external rotation than when it was subjected to internal rotation. Our study further refined the outward and inward rotation angles to quantify the change in the position of the bolt caused by ABA. This provided additional research on the relationship of the bolt to the femoral neck axis in the sagittal plane. In addition, using finite element analysis, Nan et al. (2022) reported that the stability of fracture fixation decreases when the FNS bolt is rotated forward and backward with respect to the axis of the femoral neck, which is similar to our findings. The difference is that there were fewer subgroups in Nan et al.’s study, which explored only the biomechanical changes of extreme forward and backward rotation and failed to find the changing pattern of fixation stability for different bolt forward and backward rotation angles. Nevertheless, adjusting the angle between the screw and the femoral neck axis intraoperatively is more complicated than adjusting the length of the screw or moving it up or down. In addition, repeated fluoroscopy during the operation to adjust the angle of the screw is not beneficial to patients. Therefore, if the angle of the screw placement is such that the ABA in the anteroposterior and lateral views is ≥0, no excessive adjustment is needed. This is because the overall score of the standard model (i.e., ABA = 0) remains within an acceptable range (overall score = 0.53, ranking 4th).

Although some biomechanical studies have compared the effects of different FNS positions on fixation stability (Jung et al., 2022; Nan et al., 2022; Jung et al., 2023; Kuang et al., 2023), there is still no consensus. In addition, the FNS fixation position has not been fully analyzed. For example, Jung et al. (2022) and Jung et al. (2023) reported that shortening the distance between the tip of the bolt and the subchondral bone and inserting the bolt along the femoral neck axis can help improve fixation stability. Nan et al. (2022) confirmed that in the sagittal position, a bolt should be placed in the center of the femoral neck to improve fixation stability. Kuang et al. (2023) reported that a low FNS position could achieve better fixation. However, this study comprehensively investigated the relationships between FNF stability and FNS positioning from the four angles of the bolt‒subchondral bone distance, the bolt‒femoral neck axis distance and the ABA on the AP and LAT views, compensating for the shortcomings of previous studies.

The present study is the first to comprehensively assess the biomechanical effects of FNS positioning on the stability of Pauwels type III FNFs using FEA. However, there are several limitations to this study. Although we assigned material properties to the femur using CT Hounsfield values to simulate the actual material properties of the femur as closely as possible, real bone is an anisotropic material. Second, this study explored the variation in the position of the FNS using a case study, and the conclusions still need to be verified in clinical practice with a large sample size.

## Conclusion

Our results indicate that the MIG is an important biomechanical parameter for evaluating FNS treatment of FNFs. A composite score of FNS position changes obtained using an entropy scoring method revealed the optimal fixation position under different conditions. Shortening the distance between the bolt and the subchondral bone, upward movement, external rotation and anterior rotation of the bolt can help improve the stability of the FNS for the treatment of Pauwels III FNFs.

## Data Availability

The raw data supporting the conclusions of this article will be made available by the authors, without undue reservation.

## Abbreviations

FNS, Femoral Neck System; FNF, femoral neck fracture; FEA, Finite element analysis; MIVS, the maximum implant Von-Mises stress; MISS, the maximum interfragmentary shear stress; MIG, the maximum interfragmentary gap; DHS, dynamic hip screws (DHS); CCSs, cannulated compression screws; ABA, axis-bolt angle; DICOM, digital imaging and medical communication; AP, anterior-posterior; LAT, lateral; CT, computed tomography.
