# Supplementary material for: Computational evaluation of the biomechanical effects of position changes in the femoral neck system on Pauwels type III femoral neck fractures: an in silico study
Source: Front Bioeng Biotechnol. 2025 Feb 24;13:1493555. doi: 10.3389/fbioe.2025.1493555 (PMC11891373; doi:10.3389/fbioe.2025.1493555)
Supplement: Supplementary file 1 [file DataSheet1.docx]

**Supplementary Table 1.** FEA results and composite scores for different parameters.

| **Parameters** | | **Stiffness (N/mm)** | **MIVS (MPa)** | **MISS (MPa)** | **MIG (mm)** | **Composite Score** |
| --- | --- | --- | --- | --- | --- | --- |
| **Standard model** |  | 577.13 | 67.8 | 9.7 | 1.19 | 0.53 |
| **Bolt length variation** | 75mm | 539.85 | 160.3 | 11.07 | 1.25 | 0.41 |
|  | 80mm | 573.77 | 138.4 | 10.91 | 1.22 | 0.44 |
|  | 85mm | 577.05 | 79.8 | 10.96 | 1.21 | 0.48 |
|  | 90mm | 581.72 | 106.2 | 10.94 | 1.16 | 0.51 |
| **Bolt position variation** | +5mm | 575.75 | 78.2 | 10.74 | 1.1 | 0.58 |
|  | -5mm | 544.32 | 138 | 11.02 | 1.44 | 0.24 |
|  | -10mm | 506.65 | 173 | 12.09 | 1.44 | 0.21 |
|  | -15mm | 210.22 | 268.4 | 12.93 | 1.45 | 0.19 |
| **Bolt turned outward and inward** | +3° | 437.42 | 92.5 | 9.52 | 0.98 | 0.75 |
|  | -3° | 477.27 | 94.9 | 13.22 | 1.43 | 0.23 |
|  | -6° | 433.97 | 115.1 | 15.23 | 1.43 | 0.18 |
|  | -9° | 427.93 | 152.9 | 15.55 | 1.45 | 0.13 |
| **Bolt rotation forward and backward** | +10° | 559.06 | 81.7 | 10.06 | 1.22 | 0.49 |
|  | +5° | 805.74 | 80.2 | 8.63 | 0.79 | 0.87 |
|  | -5° | 579.25 | 69.3 | 11.79 | 1.27 | 0.41 |
|  | -10° | 543.37 | 110.1 | 13.2 | 1.42 | 0.22 |

**MIVS,** the maximum implant Von-Mises stress; **MISS,** the maximum interfragmentary shear stress; **MIG,** the maximum interfragmentary gap.

**
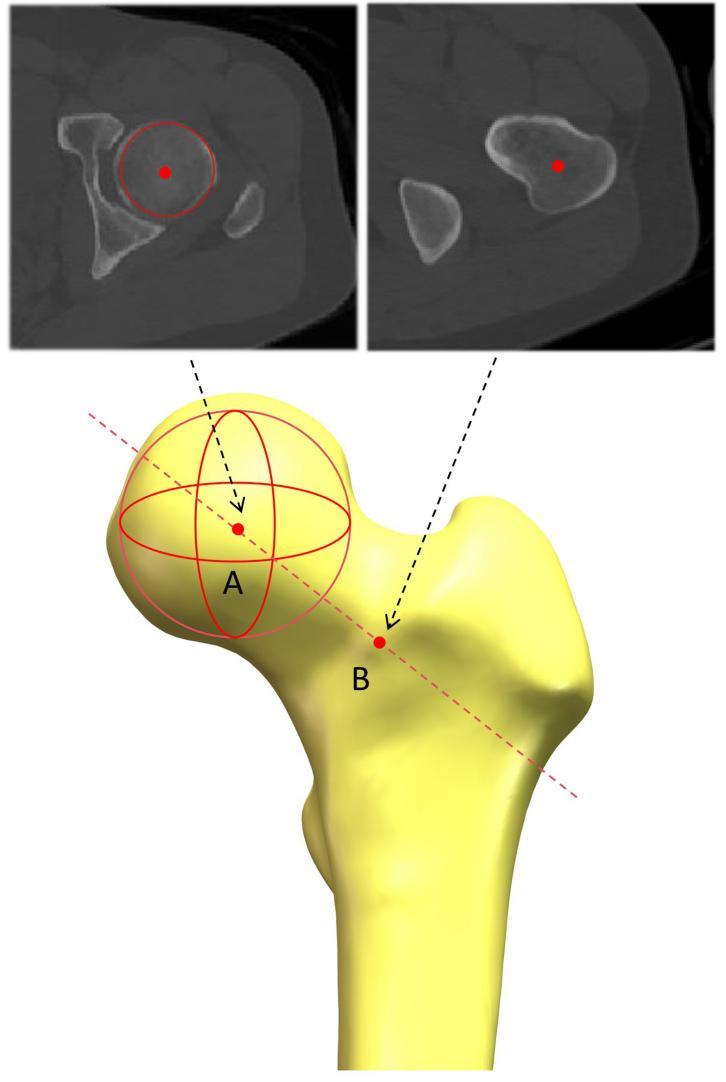
**

**Supplementary Figure 1.** Method of identifying the femoral neck axis. The upper left figure shows the maximum diameter of the femoral head in all CT planes. The upper right image shows the CT planes at the base of the femoral neck. The femoral neck axis (AB) is the line between the centre points of the two planes.


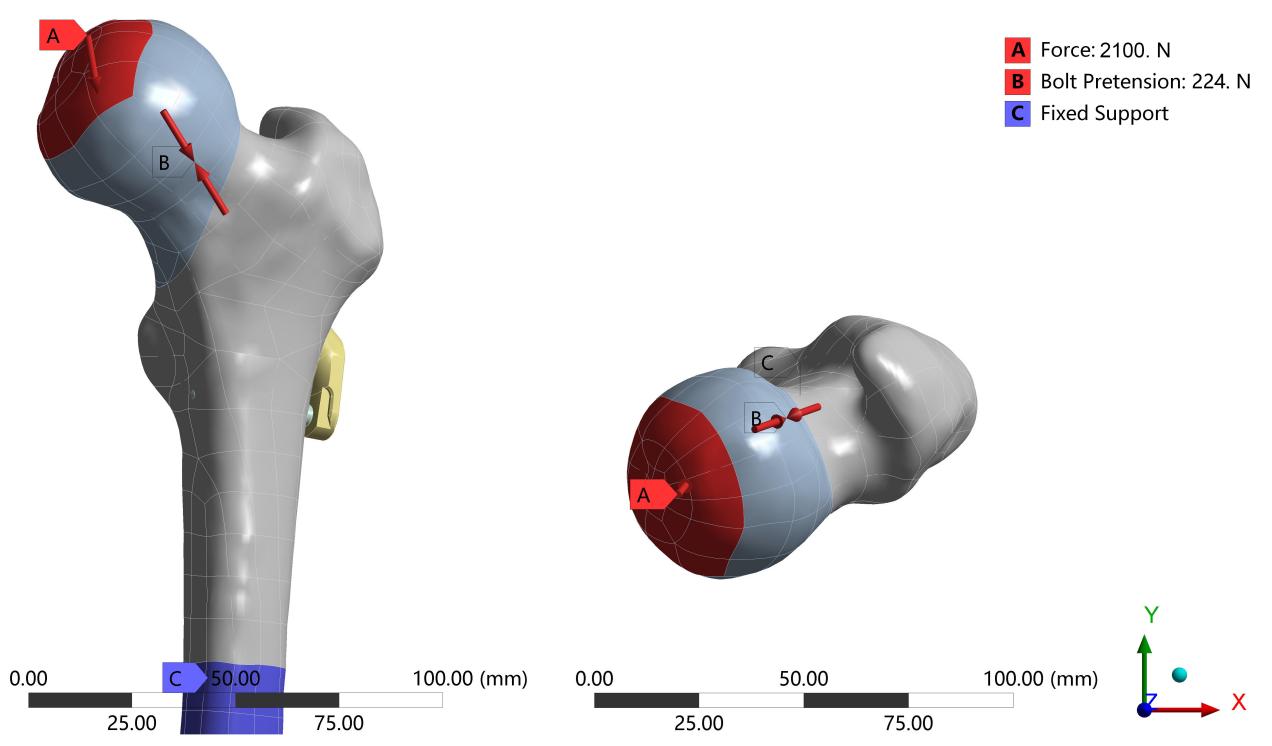


**Supplementary Figure 2.** Schematic diagram of the load applied to the femur in single-legged stance. (**A)** the femur is loaded with approximately 3 times its body weight; **(B)** the preload of the FNS anti-rotation screw; **(C)** the distal femur is fixed with a freedom of zero.
